# Semiconductor Work, Leukemia, and Cancer Risk: A Systematic Review and Meta-Analysis

**DOI:** 10.3390/ijerph192214733

**Published:** 2022-11-09

**Authors:** Kyungsik Kim, Ho Kyung Sung, Kwan Lee, Sue K. Park

**Affiliations:** 1 Department of Preventive Medicine, Seoul National University College of Medicine, Daehak-ro 101, Jongno-gu, Seoul 03080, Korea; 2 Department of Biomedical Sciences, Seoul National University Graduate School, Daehak-ro 101, Jongno-gu, Seoul 03080, Korea; 3 Cancer Research Institute, Seoul National University College of Medicine, Daehak-ro 101, Jongno-gu, Seoul 03080, Korea; 4 Institute for Public Healthcare, National Medical Center, Eulji-ro 245, Jung-gu, Seoul 04564, Korea; 5 National Emergency Medical Center, National Medical Center, Eulji-ro 245, Jung-gu, Seoul 04564, Korea; 6 Department of Preventive Medicine, Dongguk University College of Medicine, Gyeongju 38066, Korea; 7 Integrated Major in Innovative Medical Science, Seoul National University College of Medicine, Daehak-ro 101, Jongno-gu, Seoul 03080, Korea

**Keywords:** semiconductor, leukemia, cancer, meta-analysis, health assessment

## Abstract

Background: With the development of the semiconductor industry over the past 60 years, various occupational diseases have been reported to coincide with rapid industrial growth. Among these occupational diseases, the association between semiconductor work and cancers, including leukemia, remains controversial. Therefore, this systematic review and meta-analysis assesses the associations between semiconductor work, leukemia, and cancer risk. Methods: The core research databases, including PubMed, were screened for studies published until 31 July 2022. All eligible studies assessed cancer risk among workers in the semiconductor industry. Results: Nine studies were selected after a literature review. The employment period of semiconductor workers in each study was between 1965 and 2009. Semiconductor work was not significantly associated with the risk of leukemia (Relative Risk [RR], 1.02; 95% Confidence Interval [CI], 0.74–1.41) or cancer (RR, 1.00; 95% CI, 0.93–1.07). Conclusion: In this meta-analysis, semiconductor work was not significantly associated with leukemia or cancer risk. Internal comparisons, such as non-fab workers, quality of the study, employment period, and healthy worker effect, should be considered for interpretation. Furthermore, a prospective cohort study based on overall semiconductor workers in the industry could be useful to assess occupational disease risk as a mandatory component of health assessment.

## 1. Introduction

Over the last 60 years, the semiconductor industry has been developing rapidly, and is an important national strategic industry in some countries [1]. Along with the global growth of the semiconductor industry, many environmental studies have been conducted worldwide. These studies have reported associations between semiconductor work and occupational diseases, including skin problems, musculoskeletal disorders, and women’s diseases such as menstruation disorder, spontaneous abortion, and cancers [2,3,4].

Higher cancer incidence and mortality rates have been described for semiconductor workers living in the United Kingdom [5]. Several observational studies and reviews have since described an association between semiconductor work and the increased risk of several occupational diseases [6,7,8]. A previous study also reported significant rates of spontaneous abortion in female workers [9]. However, findings from existing studies are insufficient for reaching a definitive conclusion concerning the relationship between semiconductor work and occupational disease risk.

Semiconductor manufacturing processes are largely divided into three stages: (1) wafer manufacturing, (2) fabrication process, and (3) assembly. Generally, the fabrication process consists of creating a chip by engraving a semiconductor onto a wafer. Most of the previous studies defined photo-lithography, etching, clean, ion-implant, and metal processes as fabrication work; workers could be exposed to various organic solvents and occupational substances from the processes. In particular, workers may be exposed to acetone, arsenic, 2-ethoxyethanol, and dichloromethane through fabrication processes.

In the major occupational disease report in a semiconductor facility from South Korea, a female worker in her twenties died of leukemia. Subsequently, academic research needs and social interest in occupational diseases at semiconductor facility has been increasing. As a result, reports have been published concerning musculoskeletal diseases, dermatitis, cystitis, breast cancer, lymphoma, non-Hodgkin lymphoma, infertility, and ovarian cancer. Whilst all of the diseases could be related to semiconductor work, each disease can be further classified into fabrication, assembly, and overall semiconductor work-related diseases, respectively. As these previous studies primarily assessed occupational diseases according to fabrication work status, assessment of the detailed exposure source has been insufficient. Therefore, structured epidemiological studies considering fabrication processes or occupational substances are required. Bio-monitoring has recently been conducted based on biological exposure indices which consider harmful exposure levels in humans.

In this trend, a comprehensive evaluation is required through a systematic review and meta-analysis of previous studies concerning the link between occupational diseases and semiconductor work. The criteria of semiconductor workers, employment period, and comparison groups were considered as comprehensive evaluation factors. In particular, we considered leukemia, non-Hodgkin lymphoma (NHL), brain and central nervous system (CNS) cancers, breast cancer, and other cancer types.

## 2. Materials and Methods

### 2.1. Search Strategy

To identify individual studies for systematic review and meta-analysis, we used the Preferred Reporting Items for Systematic Reviews and Meta-Analyses guidelines. For the literature review, PubMed, Embase, and Cochrane Library were considered core database sources, and included studies that were published up to 31 July 2022. The search terms were “semiconductor” [MeSH Term] OR “semiconductor” [All Fields] AND “work” [MeSH Term] OR “work” [All Fields] AND “cancer” [All Fields] OR “neoplasms” [MeSH Term]. During the literature review process, Endnote X9 (Thomson Reuters, New York, NY, USA) was used for selection, which was conducted by two independent reviewers.

### 2.2. Selection Criteria

Previous studies that assessed the association between semiconductor work and cancer risk were included in this systematic review and meta-analysis. The detailed selection criteria of the studies were as follows: (1) epidemiology study dealing with cancer in semiconductor workers; (2) semiconductor work defined as: (a) overall semiconductor work, (b) fabrication (fab) work, (c) occupational substance exposure, (d) semiconductor process work; (3) comparison group, defined as general population or non-fab workers, such as office or assembly workers. For the meta-analysis, we considered (4) case-control, cohort, and cross-sectional studies; and (5) studies published in English or Korean. The exclusion criteria were in vivo or in vitro studies, exposure assessment, letters, reviews, and studies that did not assess cancer as the outcome. In addition, where there were a number of studies based on the same study population source, only one representative study was included. In this study, fabrication work is defined as a process of manufacturing semiconductor chips by engraving circuits on the wafer, as detailed processes of fabrication work, photo-lithography, diffusion, etching, ion-implant and thin film were included.

### 2.3. Quality Assessment and Data Extraction

The Newcastle-Ottawa Scale (NOS) was used in case-control and cohort studies [10]. The total NOS score was calculated for each category of the NOS tool, and all processes were conducted by independent authors. If the total score was not identical to that of the authors, another author was involved in the NOS score calculation. In the meta-analysis, a high-quality study was defined as a NOS score over 6 points; 4 and 5 points were considered as medium; and lower than 4 points were defined as low-quality studies. Detailed data extraction, first author, publication year, subject information, location, recruitment (employment) period, outcome, reported indicators, and the number of population and cases were extracted by two independent authors.

### 2.4. Statistical Analysis

For the meta-analysis, we used a random effects model based on the variance effect to estimate summary statistics. The summary statistics of each cancer, including all types of cancer, were summarized in terms of incidence and death rates. In the case of a study that described an internal comparison group, summary relative risk (RR) was presented as a summary statistic. Furthermore, the association between semiconductor work and cancer risk has been described according to the sex and quality of individual studies in subgroup analyses [11]. To present the heterogeneity, this study used both Higgins I^2^ and Cochran Q statistics [12,13]. To interpret the I^2^ test, <50%, 50–74%, and >75% were defined as low, intermediate, and high heterogeneity, respectively, and we described the detailed I^2^ statistics if it was >50% [13]. In the case of the Cochran’s Q test, a value <0.1% indicates significant heterogeneity. Furthermore, both Egger and Begg tests were conducted to assess publication bias, and a *p*-value < 0.05 defined statistically significant publication bias [14,15]. All statistical analyses were conducted using the STATA software package (version 14, StataCorp, College Station, TX, USA).

## 3. Results

**Figure 1:**
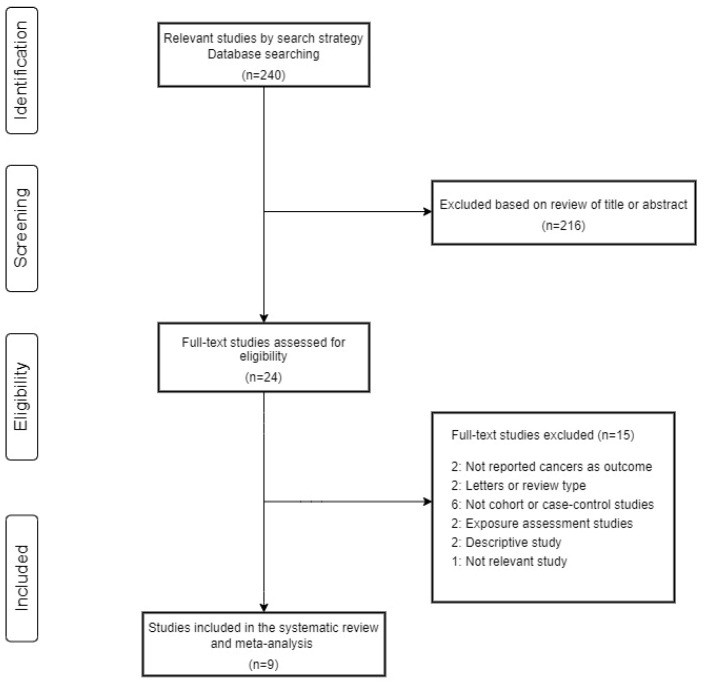
Study selection flow for systematic review and meta-analysis.

Based on the search strategy, 24 eligible full-text studies were included (Figure 1). Nine studies were included in the systematic review and meta-analysis, according to the selection criteria. The detailed characteristics of the individual studies included in the meta-analysis are described in Table 1 and Tables S1 and S2. The cancer incidence and mortality risk of semiconductor workers compared to the external comparison groups (general population) are described in Table 2. With the NOS tool, the quality of individual studies is presented in Table S3; seven studies were of low quality [5,16,17,18,19,20,21,22] and only two studies were of high quality [7,22]. Among the studies, three were conducted in Asia [7,19,21], three in Europe [5,16,17], and others in the USA [18,20,22]. When we reviewed the comparison groups, six studies used only external comparison groups [5,7,16,17,19,21], and three studies used both external and internal comparison groups [18,20,22]. The excluded studies are described in detail in Table S4.

When the general population was used as a comparison group, there were nine studies that described occupational exposure, cancer incidence rates, and mortality risk (Table 2). In the meta-analysis, we concluded that the association between semiconductor work and the risk of cancer (cancer incidence, SIR, 0.87; 95% CI, 0.82–0.92; cancer mortality, SMR, 0.70; 95% CI, 0.62–0.79), and both cancer incidence and mortality, showed intermediate and high heterogeneity, respectively (cancer incidence, I^2^, 39.1%; cancer mortality, I^2^, 72.8%). In the case of NHL, both incidence and mortality showed intermediate heterogeneity, however, neither were significantly associated with semiconductor work (NHL incidence, SIR, 1.05; 95% CI, 0.81–1.38; NHL mortality, SMR, 0.97; 95% CI, 0.68–1.39). In addition, although there was no heterogeneity in leukemia, brain and CNS, or breast cancer incidence and mortality, the association was not significant. Stratified meta-analysis, according to sex, showed that most patterns were comparable to those of overall semiconductor workers (Table 3 and Table 4). Although the incidence and mortality rates of leukemia and NHL were not significantly different between men and women, the summary point estimates appeared to be higher than those of overall semiconductor workers (female leukemia incidence, SIR, 1.17; 95% CI, 0.75–1.84; NHL incidence, SIR, 1.73; 95% CI, 0.86–3.49; female leukemia mortality, SMR, 1.27; 95% CI, 0.62–2.57; NHL mortality, SMR, 2.50; 95% CI, 0.68–6.40).

In terms of the internal comparison group-based study, there were three or four studies on each type of cancer (Table 5). Including all cancers, none of the meta-analyses were associated with fab work compared to non-fab (office or assembly) work (RR, 95% CI; all cancers, 1.00 (0.93–1.07); leukemia, 1.02 (0.74–1.41); NHL, 0.87 (0.63–1.19); brain and CNS, 0.93 (0.68–1.27); breast, 0.91 (0.64–1.31]). Regardless of the quality status, none of the meta-analyses for each cancer was associated with semiconductor work (Table 6).

## 4. Discussion

This systematic review and meta-analysis assessed cancer risk among semiconductor workers. Most of the studies included in these meta-analyses were of low quality. Although the studies were conducted in Asia, Europe, and the United States, cancer risk was comparable. In addition, most studies defined the general population as the comparison group, and only a few studies defined non-fab or office workers as the internal comparison group. Regardless of the comparison group or the quality of the studies, cancer risk was not significantly associated with semiconductor work.

It is necessary to understand the historical context of semiconductor work in order to determine its relationships with cancer risk. First, there is a lack of research on the topic. In the case of spontaneous abortion, there were only seven individual studies, including one meta-analysis [3,9,23,24]. In the case of cancer, there were at least two to six studies for each cancer type [16,17,18,19,20,21,22]. Only the United States, Taiwan, Japan, and South Korea are major semiconductor manufacturing countries, and only a few studies have been published in these countries [25]. It is essential to conduct additional studies to evaluate the health status of semiconductor workers in detail. Meanwhile, considering the major semiconductor manufacturing countries over the past 20 years, including France, Italy, Germany, and Japan, their exposure environment could be different to that of the United States, Taiwan, Japan, and South Korea. Unfortunately, exposure environment, safety management levels, and occupational substances used in the factory could not be compared between the countries. Therefore, along with the top major countries, research from other countries is needed to assess the health effect of the semiconductor workers.

Second, it is difficult to identify changes in the semiconductor work environment over time. The employment period of semiconductor workers in this study was 1970–2009, and semiconductor work was defined as fab work, overall work in the semiconductor facility, or specific processes [26]. Therefore, the assessment of detailed environmental changes is limited. In addition, owing to rapid changes in the semiconductor industry and occupational substances, it is difficult to conclude that past and current semiconductor work have the same effect on cancer risk. For example, TCE was used in the past, but it is rarely used in the current industry as a result of technological advancement and its harmful impact on humans [27]. Many alternative substances are used, however, their harmful effects on humans are insufficient [28]. According to a previous study, occupational exposure probability varies depending on the work period [29]. This suggests that direct exposure has decreased, owing to the automation system and employment change (i.e., subcontractor workers) in the semiconductor facility. However, detailed environmental changes were limited in this study.

Third, systematic exposure assessment should be considered. In South Korea, Samsung Electronics began to develop a health management system, an environmental safety integration system, and an environmentally safe workplace in 2012. Only a few studies have considered this. According to a previous study, the worker’s department, working processes, job duties, district, employment period, and use of occupational substances should be considered when assessing occupational exposure in the semiconductor industry [30]. In addition, wafer manufacturing eras and wafer size changes need to be considered as additional factors for exposure assessment [30,31]. Although most of the studies had limited information for assessing detailed occupational exposure, one study could be referred to [32], which was not included in the meta-analysis, as it used the same source population and case-control study as another study. Compared to the other studies, it dealt with various job categories and various chemicals used in the facility. Although it was impossible to evaluate the exact automatic change period, this study classified various periods in order to assess the health effect on semiconductor workers. Thus, this report on semiconductor workers is important, but a systematic consideration of the various information is required for further study.

In addition, the diversity of semiconductor employment types should be considered. In the 2000s, many semiconductor factories expanded in response to global semiconductor demand. In addition to this demand, many more semiconductor workers have been hired. In the semiconductor industry, semiconductor and subcontractor workers are employed. In the case of subcontractor workers, one of their primary duties is the prevention and maintenance of semiconductor facilities. Although most processes are automated in the current industry, there is a possibility of occupational substance exposure within these prevention and maintenance duties. Therefore, it is necessary to pay attention to the health status of subcontractor workers. However, due to the nature of their work environment and to the social issues that they face, access to individual exposure information and health status was limited. Compared to semiconductor workers, their employment period is short; therefore, access to lifetime health monitoring is limited. Therefore, a continuous study that considers various occupational substance exposure assessments are required [33,34].

Throughout the studies described in this paper, few considered multiple job categories as an exposure assessment. One of the studies described the occupational substances present in the cleanroom. Although four studies described the possible occupational substances at the facility, they did not apply them as exposure assessments. The number of subjects varied from 2000 to 130,000, and five studies consisted of more than 50,000 semiconductor workers. In the case of the employment period, four studies reported over an employment period of more than 10 years, and at least 20% to as much as 34% of workers had been working over 10 years. In addition, some studies included a study population whose employment period was less than a year. Thus, a consideration of the employment and latent period is required. Four studies could not identify the detailed age distribution; elsewhere, nearly half of the workers were younger than 30 or 40. Therefore, health effect assessment should be considered based on their age distribution.

In this meta-analysis, there were several limitations to assessing the association between semiconductor work and cancer. First, most studies defined the general population as the comparison group. As this can induce bias in assessing cancer association, considering semiconductor workers who are not exposed to occupational substances would be appropriate. In South Korea, the National Health Insurance Database can identify medical and disease history, including cancer, according to employment insurance records. Through the database, the cancer risk of semiconductor workers can be compared to various work groups. In occupational epidemiological studies, the effects on healthy worker needs to be considered. Although it is difficult to clearly identify this factor in this study, a minor impact is likely. There was no significant difference between the internal and external comparison in Table 2 and Table 5. However, given that 40% of the semiconductor workers, in several studies, were under the age of 40, careful interpretation is needed. In addition, an assessment of the association between semiconductor work and other cancer types was not available due to the lack of existing studies. Therefore, further research consideration of various cancer types is needed.

## 5. Conclusions

This systematic review and meta-analysis found no significant association between semiconductor work and cancer risk. In the evaluation of each study, it was difficult to consider changes in the semiconductor work environment and systematic exposure assessment over time. In addition, due to the inappropriate comparison group and healthy worker effect, it is difficult to conclude that semiconductor work is not a significant predictor of cancer development and mortality. Nevertheless, NHL and leukemia are still occupational diseases of interest in South Korea; therefore, lifetime monitoring is needed. As a first step, the construction of a prospective cohort, including all semiconductor workers, is essential to overcome the limitations of previous studies. Finally, this can lead to an objective and standardized health impact assessment, which can be applied in other semiconductor manufacturing countries.

## Figures and Tables

**Table 1:** General characteristics of individual studies included in the meta-analysis.

Author [Ref.]	Subjects	Location, Recruitment Period	Quality	Diseases	Reported Indicators
McElvenny, 2003 [16]	Scottish semiconductor manufacturing facility (fab and non-fab) workers	Scotland, mid-1970s–1999	6	Leukemia, Breast cancer	SIR, SMR
Darnton, 2012 [17]	Scottish semiconductor manufacturing facility (fab and non-fab) workers	Scotland, The 1970s–1999	5	Brain tumor, Breast cancer	SIR
Nichols, 2005 [5]	Semiconductor manufacturing facility workers who were employed for at least 1 month	West Midlands, UK 1970–1983	5	Leukemia, Brain tumor, Breast cancer	SIR, SMR
Bender, 2007 [18]	IBM semiconductor and electronic storage device workers	East Fishkill (NY), San Jose (CA), USA 1965–1999	5	Leukemia, NHL, Brain tumor, Breast cancer	SIR, RR
Lee, 2011 [7]	Eight Korean semiconductor manufacturing industry workers (Office and manufacturing; fabrication, assembly)	Korea, 1998–2007	7	Leukemia, NHL, Brain tumor, Breast cancer	SIR, SMR
Lee K, 2015 [19]	Samsung Electronics factories workers in two semiconductor memory facilities, 1 testing, and packaging process facility	Kiheung, Hwasung and Onyang, Korea 1998–2009	6	Leukemia, NHL	SIR
Beall, 2005 [20]	IBM employees who worked at least 1 day in two semiconductor facilities and one storage device facility	East Fishkill (NY), Burlington (VT), San Jose (CA), USA 1965–1999	5	Leukemia, NHL, Brain tumor, Breast cancer	SMR, RR
Hsieh, 2005 [21]	Eight semiconductor industry companies workers in Taiwan	Taiwan, 1980–2000	3	Leukemia	SMR
Boice, 2010 [22]	US cohort of workers involved in the manufacture of semiconductors who were employed at least 6 months	Arizona, California, New Mexico, Oregon, and Texas, USA 1983–2002	7	Leukemia, NHL, Brain tumor, Breast cancer	SMR, RR

Abbreviations: SIR, standardized incidence ratio; SMR, standardized mortality ratio; RR, relative risk; NHL, non-Hodgkin lymphoma.

**Table 2:** Meta-analysis for the standardized incidence ratios (SIRs) and standardized mortality ratios (SMRs) of cancer, leukemia, NHL, brain tumor, and breast cancer in semiconductor workers compared to the general population.

	N	Individual Studies	SIR (95% CI) ^1,4^	N	Individual Studies	SMR (95% CI) ^1,4^
Cancer					McElvenny, 2003 [16]	
					Male	0.47 (0.17–1.02)
					Female	1.10 (0.69–1.64)
		Nichols, 2005 [5]	1.00 (0.87–1.13)		Nichols, 2005 [5]	0.77 (0.63–0.92)
		Bender, 2007 [18]			Beall, 2005 [19]	0.78 (0.75–0.81)
		East Fishkill	0.81 (0.77–0.85)		Hsieh, 2005 [21]	
		San Jose	0.87 (0.82–0.92)		Male	0.41 (0.27–0.60)
		Lee, 2011 [7]			Female	0.68 (0.42–1.02)
		Male	0.86 (0.74–0.98)		Boice, 2010 [22]	0.73 (0.68–0.78)
		Female	0.88 (0.74–1.03)		Lee, 2011 [7]	
		Darnton, 2012 [17] ^1^			Male	0.44 (0.32–0.58)
		Male	0.90 (0.69–1.16)		Female	0.79 (0.51–1.18)
		Female	1.02 (0.85–1.22)			
	7	Summary SIR (95% CI)	0.87 (0.82–0.92) ^2,4^	9	Summary SMR (95% CI)	0.70 (0.62–0.79) ^2,4^
Leukemia		McElvenny, 2003 [16]	1.45 (0.04–8.06)		McElvenny, 2003 [16]	1.72 (0.04–9.61)
		Nichols, 2005 [5]	1.21 (0.39–2.83)		Nichols, 2005 [5]	0.96 (0.20–2.82)
		Bender, 2007 [18]			Beall, 2005 [19]	0.85 (0.69–1.05)
		East Fishkill	0.70 (0.49–0.98)		Hsieh, 2005 [21]	2.18 (0.87–4.49)
		San Jose	1.03 (0.73–1.42)		Lee, 2011 [7]	
		Lee, 2011 [7]			Male	0.39 (0.08–1.14)
		Male	0.69 (0.30–1.37)		Female	1.37 (0.55–2.81)
		Female	1.28 (0.61–2.36)		Boice,2010 [22]	0.77 (0.54–1.07)
		Lee K, 2015 [19]	0.86 (0.50–1.47)			
	7	Summary SIR (95% CI)	0.89 (0.73–1.08) ^3,4^	7	Summary SMR (95% CI)	0.92 (0.71–1.20) ^3,4^
NHL		Bender, 2007 [18]				
		East Fishkill	0.94 (0.74–1.18)		Beall, 2005 [20]	0.99 (0.82–1.19)
		San Jose	0.91 (0.69–1.17)		Boice, 2010 [22]	0.69 (0.48–0.97)
		Lee, 2011 [7]			Lee, 2011 [7]	
		Male	0.93 (0.45–1.71)		Male	1.33 (0.43–3.09)
		Female	2.31 (1.23–3.95)		Female	2.50 (0.68–6.40)
		Lee K, 2015 [19]	0.93 (0.51–1.67)			
	5	Summary SIR (95% CI)	1.05 (0.81–1.38) ^2,4^	4	Summary SMR (95% CI)	0.97 (0.68–1.39) ^2,3,4^
Brain		Nichols, 2005 [5]	0.50 (0.06–1.81)		Nichols, 2005 [5]	0.83 (0.17–2.43)
		Bender, 2007 [18]	CNS		Beall, 2005 [20]	1.08 (0.87–1.32)
		East Fishkill	0.94 (0.65–1.32)		Boice, 2010 [22]	1.11 (0.84–1.45)
		San Jose	0.91 (0.56–1.39)		Lee, 2011 [7]	
		Lee, 2011 [7]			Male	0.92 (0.25–2.35)
		Male	1.37 (0.62–2.59)		Female	0.34 (0.01–1.87)
		Female	0.22 (0.01–1.22)			
		Darnton, 2012 [17]	2.09 (0.57–5.35)			
	6	Summary SIR (95% CI)	0.99 (0.77–1.26) ^3,4^	5	Summary SMR (95% CI)	1.08 (0.92–1.27) ^3,4^
Breast					McElvenny, 2003 [16]	0.74 (0.20–1.90)
		Nichols, 2005 [5]	0.78 (0.59–1.02)		Nichols, 2005 [5]	0.47 (0.25–0.81)
		Bender, 2007 [18]			Beall, 2005 [20]	0.95 (0.80–1.12)
		East Fishkill	1.04 (0.89–1.20)		Boice, 2010 [22]	0.92 (0.75–1.12)
		San Jose	1.02 (0.87–1.19)		Lee, 2011 [7]	0.84 (0.10–3.02)
		Lee, 2011 [7]	0.77 (0.44–1.26)			
		Darnton, 2012 [17]	1.22 (0.90–1.63)			
	5	Summary SIR (95% CI)	1.00 (0.87–1.13) ^3,4^	5	Summary SMR (95% CI)	0.88 (0.74–1.05) ^3,4^

Abbreviation: SIR, standardized incidence ratio; SMR, standardized mortality ratio. ^1^ Heterogeneity and publication bias across studies were presented only when the number of individual studies was 5 or more. ^2^ Heterogeneity: I^2^ = 39.1% (54.3% (NHL) for SIR; I^2^ = 72.8%, 55.0% (NHL) for SMR. ^3^ No heterogeneity I^2^ < 50% regardless of Cochran *p*-value. ^4^ No publication bias in Begg or Egger test; *p* > 0.05.

**Table 3:** Sex-specific meta-analysis for the standardized incidence ratios (SIRs) of cancer, leukemia, NHL, and brain tumor in semiconductor workers compared to the general population.

Study N		SIR (95% CI) ^1^	Study N		SIR (95% CI) ^1^
	Men			Women	
	Cancer			Cancer	
	Nichols, 2005 [5]	1.30 (0.95–1.73)		Nichols, 2005 [5]	0.94 (0.82–1.09)
	Lee, 2011 [7]	0.86 (0.74–0.98)		Lee, 2011 [7]	0.88 (0.74–1.03)
	Darnton, 2012 [17]	0.90 (0.69–1.16)		Darnton, 2012 [17]	1.02 (0.85–1.22)
3	Summary SIR (95% CI)	0.98 (0.78–1.23)	3	Summary SIR (95% CI)	0.94 (0.86–1.03)
	Leukemia			Leukemia	
	Nichols, 2005 [5]	2.33 (0.28–8.40)		McElvenny, 2003 [16] ^1^	1.45 (0.04–8.06)
	Lee, 2011 [7]	0.69 (0.30–1.37)		Nichols, 2005 [5]	0.91 (0.19–2.67)
	Lee K, 2015 [19]	0.65 (0.27–1.57)		Lee, 2011 [7]	1.28 (0.61–2.36)
				Lee K, 2015 [19]	1.13 (0.56–2.26)
3	Summary SIR (95% CI)	0.76 (0.44–1.32)	4	Summary SIR (95% CI)	1.17 (0.75–1.84)
	NHL			NHL	
	Lee, 2011 [7]	0.93 (0.45–1.71)		Lee, 2011 [7]	2.31 (1.23–3.95)
	Lee K, 2015 [19]	0.83 (0.37–1.85)		Lee K, 2015 [19]	1.11 (0.46–2.67)
2	Summary SIR (95% CI)	0.89 (0.531.48)	2	Summary SIR (95% CI)	1.73 (0.86–3.49)
	Brain			Brain	
	Lee, 2011 [7]	1.37 (0.62–2.59)		Nichols, 2005 [5]	0.61 (0.07–2.21)
				Lee, 2011 [7]	0.22 (0.01–1.22)
1	Summary SIR (95% CI)	1.37 (0.62–2.59)	2	Summary SIR (95% CI)	0.43 (0.11–1.75)

^1^ Heterogeneity and publication bias across studies were presented only when the number of individual studies was 5 or more.

**Table 4:** Sex-specific meta-analysis for the standardized mortality ratios (SMRs) of cancer, leukemia, NHL, and brain tumor in semiconductor workers compared to the general population.

Study N		SMR (95% CI) ^1^	Study N		SMR (95% CI) ^1^
	Men			Women	
	Cancer			Cancer	
	McElvenny, 2003 [16] ^1^	0.47 (0.17–1.02)		McElvenny, 2003 [16] ^1^	1.10 (0.69–1.64)
	Nichols, 2005 [5]	1.12 (0.75–1.61)		Nichols, 2005 [5]	0.69 (0.55–0.86)
	Hsieh, 2005 [21]	0.41 (0.27–0.60)		Hsieh, 2005 [21]	0.68 (0.42–1.02)
	Lee, 2011 [7]	0.44 (0.32–0.58)		Lee, 2011 [7]	0.79 (0.51–1.18)
4	Summary SMR (95% CI)	0.56 (0.33–0.95)	4	Summary SMR (95% CI)	0.77 (0.63–0.94)
	Leukemia			Leukemia	
	Nichols, 2005 [5]	1.59 (0.08–7.83)		McElvenny, 2003 [16] ^1^	1.72 (0.04–9.61)
	Hsieh, 2005 [21]	2.18 (0.87–4.49)		Nichols, 2005 [5]	0.80 (0.10–2.91)
	Lee, 2011 [7]	0.39 (0.08–1.14)		Lee, 2011 [7]	1.37 (0.55–2.81)
3	Summary SMR (95% CI)	1.13 (0.34–3.78)	3	Summary SMR (95% CI)	1.27 (0.62–2.57)
	NHL			NHL	
	Lee, 2011 [7]	1.33 (0.43–3.09)		Lee, 2011 [7]	2.50 (0.68–6.40)
1	Summary SMR (95% CI)	1.33 (0.43–3.09)	1	Summary SMR (95% CI)	2.50 (0.68–6.40)
	Brain			Brain	
	Lee, 2011 [7]	0.92 (0.25–2.35)		Nichols, 2005 [5]	1.02 (0.21–2.98)
				Lee, 2011 [7]	0.34 (0.01–1.87)
1	Summary SMR (95% CI)	0.92 (0.25–2.35)	2	Summary SMR (95% CI)	0.82 (0.25–2.66)

^1^ Heterogeneity and publication bias across studies were presented only when the number of individual studies was 5 or more.

**Table 5:** Meta-analysis for the relative risks (RRs) of cancer, leukemia, NHL, central nervous system cancers, and breast cancer in exposed semiconductor workers compared to non-exposed semiconductor workers.

	Studies N	Individual Studies	RR (95% CI) in Each Study
Cancer	3	Bender, 2007 [18]	
		East Fishkill	1.0 (0.9–1.2)
		San Jose	1.0 (0.9–1.1)
		Boice, 2010 [22]	0.98 (0.8–1.1)
		Summary RR (95% CI)	1.00 (0.93–1.07) ^1,2^
Leukemia	4	Bender, 2007 [18]	
		East Fishkill	1.1 (0.5–2.4)
		San Jose	1.1 (0.5–2.2)
		Boice, 2010 [22]	0.96 (0.5–1.9)
		Beall, 2005 [20]	1.0 (0.6–1.6)
		Summary RR (95% CI)	1.02 (0.74–1.41) ^1,2^
NHL	4	Bender, 2007 [18]	
		East Fishkill	1.2 (0.6–2.2)
		San Jose	0.7 (0.4-.1.1)
		Boice, 2010 [22]	1.34 (0.7–2.6)
		Beall, 2005 [20]	0.7 (0.5–1.0)
		Summary RR (95% CI)	0.87 (0.63–1.19) ^1,2^
Brain	4	Bender, 2007 [18]	
		East Fishkill	1.2 (0.5–3.0)
		San Jose	0.8 (0.3–1.9)
		Boice, 2010 [22]	0.76 (0.4–1.4)
		Beall, 2005 [20]	1.0 (0.7–1.7)
		Summary RR (95% CI)	0.93 (0.68–1.27) ^1,2^
Breast	4	Bender, 2007 [18]	
		East Fishkill	0.8 (0.6–1.1)
		San Jose	1.2 (0.8–1.6)
		Boice, 2010 [22]	0.62 (0.4–1.0)
		Beall, 2005 [20]	0.8 (0.6–1.2)
		Summary RR (95% CI)	0.91 (0.64–1.31) ^1,2^

^1^ Heterogeneity and publication bias across studies were presented only when the number of individual studies was 5 or more. ^2^ No heterogeneity I^2^ < 50% regardless of Cochran *p*-value.

**Table 6:** Sub-group meta-analysis by study quality for the standardized incidence ratios (SIRs) and standardized mortality ratios (SMRs) of cancer, leukemia, NHL, brain tumor and breast cancer in semiconductor workers compared to the general population.

Cancer Type	Quality	Study N	Summary SIR (95% CI)	Study N	Summary SMR (95% CI) ^1^
Total cancers	High quality	4	0.91 (0.83–0.99)	5	0.69 (0.51–0.91) ^2,3^
	Middle quality	3	0.87 (0.80–0.96) ^2^	2	0.78 (0.75–0.81)
Leukemia	High quality	4	0.93 (0.64–1.34)	4	0.82 (0.58–1.15)
	Middle quality	2	0.88 (0.65–1.20)	2	0.85 (0.69–1.05)
NHL	High quality	3	1.27 (0.69–2.34)	3	1.14 (0.53–2.49)
	Middle quality	2	0.92 (0.77–1.10)	1	0.99 (0.82–1.19)
Brain tumor	High quality	3	1.33 (0.61–2.89)	3	1.09 (0.83–1.41)
	Middle quality	3	0.91 (0.69–1.20)	2	1.07 (0.87–1.32)
Breast cancer	High quality	2	1.02 (0.66–1.59)	3	0.91 (0.75–1.11)
	Middle quality	3	0.98 (0.85–1.12)	2	0.71 (0.36–1.40)

Abbreviation: NHL, Non-Hodgkin’s lymphoma; SIR, standardized incidence ratio; SMR, standardized mortality ratio. ^1^ Heterogeneity and publication bias across studies were presented only when the number of individual studies was 5 or more. ^2^ Heterogeneity: I^2^ = 76.2% for SMR. ^3^ No publication bias in Begg or Egger test; *p* > 0.05.

## Data Availability

No new data were created or analyzed in this study. Data sharing is not applicable to this article.

## Supplementary Materials

The following supporting information can be downloaded at: https://www.mdpi.com/article/10.3390/ijerph192214733/s1, Table S1: Existing studies on the risk of cancer of semiconductor workers compared to external comparison groups (general population). Table S2: Existing studies on the risk of cancer and specific cancers (leukemia, NHL, brain tumor and female breast cancer) of semiconductor exposed workers compared to internal comparison groups (office work or non–fabrication work). Table S3: Results of literature quality evaluation using the Newcastle Ottawa Scale (NOS). Table S4: The excluded studies during the systematic review and meta-analysis.
