# Supplementary material for: Semiconductor Work, Leukemia, and Cancer Risk: A Systematic Review and Meta-Analysis
Source: Int J Environ Res Public Health. 2022 Nov 9;19(22):14733. doi: 10.3390/ijerph192214733 (PMC9690168; doi:10.3390/ijerph192214733)
Supplement: Supplementary file 1 [file ijerph-19-14733-s001.zip › ijerph-1963312-supplementary.pdf]

Table S1. Existing studies on the risk of cancer of semiconductor workers compared to external comparison groups (general population)

Author [Ref.]	Outcome	Group	Cohort	Event	SIR (95% CI)	Cohort	Event	SMR (95% CI)
			N	N		N	N	
McElvenny, 2003[16] [1]	Cancer	Total	4,388	79	1.07 (0.85–1.35) [2]	4,388	29	0.94 (0.63–1.38) [2]
		Male	2,126	25	0.99 (0.64–1.47)	2,126	6	0.47 (0.17–1.02)
		Female	2,262	54	1.11 (0.83–1.45)	2,262	23	1.10 (0.69–1.64)
	Leukemia	Male	2,126	0	0 (0–5.43)	2,126	0	0 (0–0.81)
		Female	2,262	1	1.45 (0.04–8.06)	2,262	1	1.72 (0.04–9.61)
	Breast	Female	2,262	20	1.34 (0.82–2.06)	2,262	4	0.74 (0.20–1.90)
Nichols, 2005 [5]	Cancer	Total	1,807	239	1.00 (0.87–1.13)	1,807	111	0.77 (0.63–0.92)
		Male	281	46	1.30 (0.95–1.73)	281	29	1.12 (0.75–1.61)
		Female	1,526	193	0.94 (0.82–1.09)	1,526	82	0.69 (0.55–0.86)
	Leukemia	Total	1,807	5	1.21 (0.39–2.83)	1,807	3	0.96 (0.20–2.82)
		Male	281	2	2.33 (0.28–8.40)	281	1	1.59 (0.08–7.83)
		Female	1,526	3	0.91 (0.19–2.67)	1,526	2	0.80 (0.10–2.91)
	Brain	Total	1,807	2	0.50 (0.06–1.81)	1,807	3	0.83 (0.17–2.43)
		Female	1,526	2	0.61 (0.07–2.21)	1,526	3	1.02 (0.21–2.98)
	Breast	Total	1,807	54	0.78 (0.59–1.02)	1,807	13	0.47 (0.25–0.81)
Beall, 2005 [20]	Leukemia	Total				126,836	91	0.85 (0.69–1.05)
		Exposed [1]				90,756	63	0.86 (0.66–1.09)
	NHL	Total				126,836	116	0.99 (0.82–1.19)
		Exposed [1]				90,756	73	0.90 (0.70–1.13)
	Breast	Total				126,836	137	0.95 (0.80–1.12)
		Exposed [1]				90,756	74	0.86 (0.67–1.08)
Hsieh, 2005 [21]	Cancer	Total				47,426	50	0.51 (0.38–0.69) [2]
		Male				19,816	27	0.41 (0.27–0.60)
		Female				27,610	23	0.68 (0.42–1.02)
	Leukemia	Male				19,816	7	2.18 (0.87–4.49)
Bender, 2007 [18]	Cancer	Total	89,054	2,860	0.84 (0.80–0.87) [3]			
		East Fishkill			0.81 (0.77–0.85)			
		San Jose			0.87 (0.82–0.92)			
		Exposed	62,819	1,752	0.85 (0.81–0.90) [3]			
		East Fishkill			0.82 (0.77–0.87)			
		San Jose			0.92 (0.85–1.00)			
	Leukemia	Total	89,054	72	0.86 (0.67–1.09) [3]			
		East Fishkill			0.70 (0.49–0.98)			
		San Jose			1.03 (0.73–1.42)			
		Exposed	62,819	50	0.84 (0.63–1.13) [3]			
		East Fishkill			0.70 (0.46–1.02)			
		San Jose			1.04 (0.66–1.56)			
	NHL	Total	89,054	134	0.94 (0.75–1.17) [3]			
		East Fishkill			0.94 (0.74–1.18)			
		San Jose			0.91 (0.69–1.17)			
		Exposed	62,819	91	0.91 (0.73–1.12) [3]			
		East Fishkill			1.00 (0.76–1.28)			

		San Jose			0.74 (0.50–1.06)			
	Brain	Total	89,054	55	0.93 (0.70–1.23) [3]			
		East Fishkill			0.94 (0.65–1.32)			
		San Jose			0.91 (0.56–1.39)			
		Exposed	62,819	40	0.97 (0.69–1.34) [3]			
		East Fishkill			1.01 (0.67–1.47)			
		San Jose			0.87 (0.45–1.52)			
	Breast	Total	89,054	347	1.03 (0.93–1.15) [3]			
		East Fishkill			1.04 (0.89–1.20)			
		San Jose			1.02 (0.87–1.19)			
		Exposed	62,819	193	0.98 (0.85–1.13) [3]			
		East Fishkill			0.98 (0.81–1.17)			
		San Jose			0.97 (0.77–1.21)			
Boice,2010 [22]	Cancer	Total				100,081	832	0.73 (0.68–0.78)
		Fab. work				37,225	290	0.74 (0.66–0.83)
	Leukemia	Total				100,081	35	0.77 (0.54–1.07)
		Fab. work				37,225	11	0.69 (0.34–1.23)
	NHL	Total				100,081	33	0.69 (0.48–0.97)
		Fab. work				37,225	14	0.86 (0.47–1.44)
	Brain	Total				100,081	54	1.11 (0.84–1.45)
		Fab. work				37,225	14	0.83 (0.45–1.39)
	Breast	Total				100,081	101	0.92 (0.75–1.12)
		Fab. work				37,225	26	0.65 (0.42–0.95)
Lee, 2011 [7]	Cancer	Total	108,933	346	0.87 (0.78–0.97) [2]	113,443	72	0.54 (0.42–0.68) [2]
		Male	46,826	201	0.86 (0.74–0.98)	48,589	48	0.44 (0.32–0.58)
		Female	62,107	145	0.88 (0.74–1.03)	64,854	24	0.79 (0.51–1.18)
	Leukemia	Total	108,933	18	0.97 (0.59–1.61) [2]	113,443	10	0.97 (0.49–1.95) [2]
		Fab. work, total	78,079	13	1.01 (0.55–1.85) [2]			
		Male	46,826	8	0.69 (0.30–1.37)	48,589	3	0.39 (0.08–1.14)
		Fab. work, male	32,037	6	0.80 (0.29–1.75)			
		Female	62,107	10	1.28 (0.61–2.36)	64,854	7	1.37 (0.55–2.81)
		Fab. work, female	46,042	7	1.22 (0.49–2.52)			
	NHL	Total	108,933	23	1.56 (1.00–2.42) [2]	113,443	9	1.75 (0.84–3.67) [2]
		Fab. work, total	78,079	16	1.54 (0.90–2.64) [2]			
		Male	46,826	10	0.93 (0.45–1.71)	48,589	5	1.33 (0.43–3.09)
		Fab. work, male	32,037	8	1.21 (0.52–2.38)			
		Female	62,107	13	2.31 (1.23–3.95)	64,854	4	2.50 (0.68–6.40)
		Fab. work, female	46,042	8	1.96 (0.85–3.86)			
	Brain	Total	108,933	10	1.18 (0.60–2.34) [2]	113,443	5	0.79 (0.28–2.21) [2]
		Male	46,826	9	1.37 (0.62–2.59)	48,589	4	0.92 (0.25–2.35)
		Female	62,107	1	0.22 (0.01–1.22)	64,854	1	0.34 (0.01–1.87)
	Breast	Female	62,107	16	0.77 (0.44–1.26)	64,854	2	0.84 (0.10–3.02)
Darnton, 2012 [17]	Cancer	Total	4,388	184	0.98 (0.84–1.14) [2]			
		Male		62	0.90 (0.69–1.16)			
		Female		122	1.02 (0.85–1.22)			

	Brain			4	2.09 (0.57–5.35)
	Breast			46	1.22 (0.90–1.63)
Lee K, 2015 [19]	Leukemia	Total	56,283	13	0.86 (0.50–1.47)
		Fab. work	35,491	NR	0.95 (0.49–1.82)
		Male	24,820	5	0.65 (0.27–1.57)
		Fab. work	15,029	NR	0.66 (0.21–2.05)
		Female	31,463	8	1.13 (0.56–2.26)
		Fab. work	20,462	NR	1.30 (0.59–2.90)
	NHL	Total	56,283	11	0.93 (0.51–1.67)
		Fab. work	35,491	NR	0.55 (0.21–1.48)
		Male	24,820	6	0.83 (0.37–1.85)
		Fab. work	15,029	NR	0.96 (0.36–2.56)
		Female	31,463	5	1.11 (0.46–2.67)

Abbreviations: SIR; standardized incidence ratio, SMR; standardized mortality ratio, NR; not reported; Fab, Fabrication

1. 'Exposed' means exposed workers entailed any type of exposure work other than office work

2. Summary SIR (95% CI) using two SIRs or SMRs for men and women.

3. Summary SIR (95% CI) using two SIRs or SMRs from East Fishkill and San Jose.

Table S2. Existing studies on the risk of cancer and specific cancers (leukemia, NHL, brain tumor and female breast cancer) of semiconductor exposed workers compared to internal comparison groups (office work or non–fabrication work)

Author [Ref.]	Exposure vs. Unexposed groups	Outcome	Event N	RR (95% CI)
				**Cancer incidence**
Bender, 2007 [18]	Manufacturing work	Cancer	1,752	1.00 (0.92–1.09)[1]
	Vs. Office work	East Fishkill		1.0 (0.9–1.2)
	Total cohort N=89,054	San Jose		1.0 (0.9–1.1)
		Leukemia	50	1.10 (0.64–1.89) [1]
		East Fishkill		1.1 (0.5–2.4)
		San Jose		1.1 (0.5–2.2)
		NHL	91	1.20 (0.6–1.94) [1]
		East Fishkill		0.7 (0.4-1.1)
		San Jose		1.2 (0.6–2.2)
		Brain	40	0.99 (0.52–1.88) [1]
		East Fishkill		1.2 (0.5–3.0)
		San Jose		0.8 (0.3–1.9)
		Breast	193	0.95 (0.76–1.12) [1]
		East Fishkill		0.8 (0.6-1.1)
		San Jose		1.2 (0.8-1.6)
				**Cancer death**
Boice,2010 [22]	Fabrication work	Cancer	312	0.98 (0.80–1.10)
	Vs. Non–fabrication work	Leukemia	13	0.96 (0.50–1.90)
	Total cohort N=100,081	NHL	18	1.34 (0.70–2.60)
		Brain	15	0.76 (0.40–1.40)
		Breast	27	0.62 (0.40–1.00)
Beall,2005 [20]	Manufacturing work	Leukemia	63	1.00 (0.60–1.60)
	Vs. Office work	NHL	73	0.70 (0.50–1.00)
	Total cohort N=126,836	Brain	68	1.00 (0.70–1.70)
		Breast	74	0.80 (0.60–1.20)

Abbreviation, RR, relative risk

1. Summary RR (95% CI) using two RRs from East Fishkill and San Jose.

Table S3. Results of literature quality evaluation using the Newcastle Ottawa Scale (NOS)

	Quality evaluation criteria in cohort study									
	Selection				Comparability		Outcome			
Authors, Year	Exposed cohort	Non–exposed cohort	Ascertain ment of exposure	Outcome of interest	Comparability of the design or controlled for confounders		Assessment of outcome	Length of follow–up	Adequacy of follow–up	NOS score
McElvenny et al, 2003 [16]	○	–	○	–	○	–	○	○	○	6
Beall et al, 2005 [20]	○	–	○	–	–	–	○	○	○	5
Nichols et al, 2005 [5]	○	–	–	–	○	–	○	○	○	5
Bender et al, 2007 [18]	○	–	○	–	–	–	○	○	○	5
Boice et al, 2010 [22]	○	–	○	–	○	○	○	○	○	7
Lee et al, 2011 [7]	○	–	○	○	○	–	○	○	○	7
Darnton et al, 2012 [17]	○	–	○	–	○	–	○	○	○	6
Lee K et al, 2015 [19]	○	–	○	–	○	–	○	○	○	6
Hsieh et al, 2005 [21]	–	–	○	–	–	–	○	–	–	2

Table S4. The excluded studies during the systematic review and meta-analysis

[Not reported cancers as outcome]

1. Semiconductor Work and the Risk of Spontaneous Abortion: A Systematic Review and Meta-Analysis
2. Occupational lung diseases in the industrializing and industrialized world due to modern industries and modern pollutants

[Not cohort or case-control studies]

1. Occupational Characteristics of Semiconductor Workers with Cancer and Rare Diseases Registered with a Workers' Compensation Program in Korea
2. Health Status, Health-Related Factors and Work Environment in Korean Semiconductor Workers between 1984-2012: A Qualitative Study and a Cross-Sectional Study
3. Cases series of malignant lymphohematopoietic disorder in korean semiconductor industry
4. Cancer risks for humans from exposure to the semiconductor metals.
5. Application of the adverse outcome pathway framework to predict the toxicity of chemicals in the semiconductor manufacturing industry
6. Review for Retrospective Exposure Assessment Methods Used in Epidemiologic Cancer Risk Studies of Semiconductor Workers: Limitations and Recommendations

[Letters or review type]

1. Two Semiconductor Companies' Financial Support Compensation (FSC) Programs for Semiconductor Workers with Suspected Work-Related Diseases (WRDs)
2. Cancer risk in the semiconductor industry: Responding to the call for action

[Descriptive study]

1. Case-control and case-only studies of selected cancers at a Scottish semiconductor manufacturing facility (Abstarct only)
2. Health and safety executive inspection of U.K. semiconductor manufacturers.

[Exposure assessment]

1. Exposure assessment for retrospective follow-up studies of semiconductor- and storage device-manufacturing workers
2. Biological Assessment of Potential Exposure to Occupational Substances in Current Semiconductor Workers with at Least 5 Years of Employment

[Not relevant study]

1. Case-control study of brain and other central nervous system cancer among workers at semiconductor and storage device manufacturing facilities (Same source of East Fishkill, Burlington and San Jose)
